# Thermo-Oxidation of Phytosterol Molecules in Rapeseed Oil during Heating: The Impact of Unsaturation Level of the Oil

**DOI:** 10.3390/foods10010050

**Published:** 2020-12-26

**Authors:** Dominik Kmiecik, Monika Fedko, Magdalena Rudzińska, Aleksander Siger, Anna Gramza-Michałowska, Joanna Kobus-Cisowska

**Affiliations:** 1 Department of Gastronomy Science and Functional Food, Faculty of Food Science and Nutrition, Poznan University of Life Sciences, Wojska Polskiego 31, 60-634 Poznan, Poland; monika.fedko@up.poznan.pl (M.F.); anna.gramza@up.poznan.pl (A.G.-M.); joanna.kobus-cisowska@up.poznan.pl (J.K.-C.); 2 Department of Food Technology of Plant Origin, Faculty of Food Science and Nutrition, Poznan University of Life Sciences, Wojska Polskiego 31, 60-634 Poznan, Poland; magdalena.rudzinska@up.poznan.pl; 3 Department of Biochemistry and Food Analysis, Faculty of Food Science and Nutrition, Poznan University of Life Sciences, Wojska Polskiego 31, 60-634 Poznan, Poland; aleksander.siger@up.poznan.pl

**Keywords:** phytosterols, oxyphytosterols, phytosterols oxidation product, heating process, pressed, refined and partially hydrogenated rapeseed oil

## Abstract

Phytosterols are naturally occurring substances in foods of plant origin that have positive effects on the human body. Their consumption can reduce the level of low density lipoprotein (LDL) cholesterol. The presence of unsaturated bonds in their structure leads to their oxidation during production, storage, and thermal processes. The aim of the study was to determine how the degree of unsaturation of rapeseed oil affects the oxidation of phytosterols in oil during 48 h of heating. In all not-heated oils, the dominant groups of oxyphytosterols were 7α- and 7β-hydroxy sterols. During 48 h of heating, the rapid decrease of phytosterols’ levels and the increase of the content of oxyphytosterols were observed. The main dominant group in heated samples was hydroxy and epoxy sterols. Despite differences in fatty acid composition and content and composition of single phytosterols in unheated oils samples, the total content of oxyphytosterols after finishing of heating was on a similar level for each of the tested oils. This showed that the fatty acid composition of oil is not the only factor that affects the oxidation of phytosterols in foods during heating.

## 1. Introduction

Frying is one of the most popular food-cooking methods used in household kitchens, fast-food restaurants, and industry. Fried food is tasty, flavorful, and has a unique, characteristic structure. The frying process is also cheap and very fast [1]. Oil used in deep-fat frying processes is an excellent heat-transfer medium; thus, the food is quickly heated and cooked when it is immersed into the oil [2]. However, the high temperature of the frying process (usually 170–180 °C), long time of use oil, and other process conditions (type of frying food and oil, and frying method) affect the degradation of frying oil [3,4,5,6]. The typical chemical reactions observed during deep-fat frying can be classified as hydrolysis, oxidation, isomerization, and polymerization [7]. Oxidation can be classified as one of the most important reaction influences on the oil quality and its nutritional value. The lipid oxidation caused by atmospheric oxygen and heat leads to the formation of rancid odors and flavors in frying food and decrease in the shelf life of oil; it also decreases the nutritional value and safety via the formation of potentially toxic secondary compounds [8]. The oxidation process is not only typical for unsaturated fatty acid but also for other oil compounds, such as tocopherols and phytosterols [9,10,11,12].

**Figure 1:**
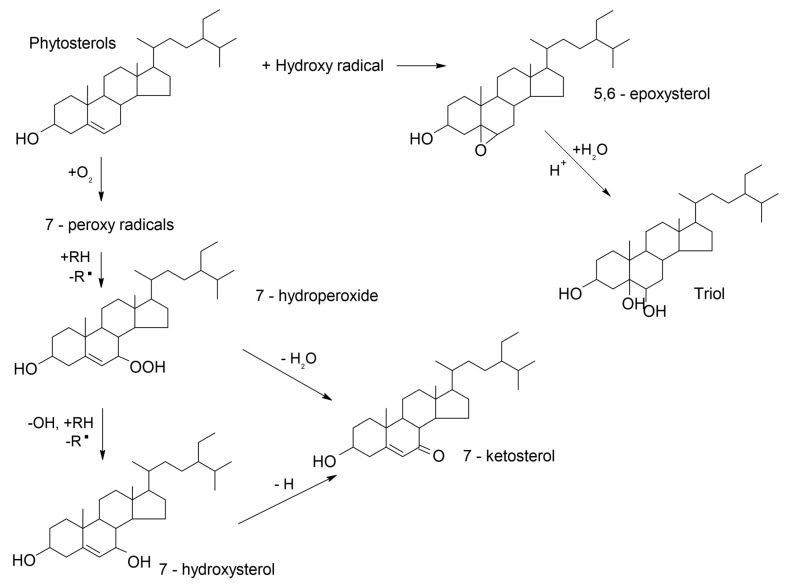
Scheme of phytosterol oxidation. Adapted from References [18,19].

Phytosterols are substances present in all plant food, but the main sources of these compounds in the human diet are oils such as rice bran, wheat germ, corn, rapeseed, and sunflower oil. Additionally, good sources of plant sterols can be foods enriched with phytosterols such as margarine, fermented milk drinks, milk, cheesy products, chocolate, or ready-to-eat meals [13]. Presence of unsaturated bonds in structure of phytosterols leads to their oxidation and formation of phytosterols oxidation products (POPs), such as 7 or 25-hydroxyphytosterols, 7 ketophytosterols, epoxyphytosterols, or phytosterotriol (Figure 1). During the last few years, researchers have observed the formation of POPs in all steps of production and storage of oil, frying foods, and foods enriched with phytosterols [14,15,16]. The presence of POPs in food is undesired due to their potential toxic effects; however, despite the similarity in structure between POPs and cholesterol oxidation products (COPs), their mechanism is still not fully understood [17].

The use of oil (natural source of phytosterols) in the frying process leads to their degradation. The amount of POP formed during frying mostly depends on the type of oil (amount of phytosterols, antioxidant content, and degree of unsaturation), the process conditions (temperature and time), and on the fried food (food composition).

The way to reduce the oxidation during frying may be the use of oils with higher oxidative stability (low content of polyunsaturated fatty acids and high concentration of natural antioxidants) and addition of antioxidants. Oils with a low content of polyunsaturated fatty acids are, for example, olive oils, and also modified oils, such as high oleic sunflower oil, high oleic rapeseed oil, or high oleic soybean oil. In this oil, the fatty acid profile was changed by increasing the presence of oleic acid (18:1) and minimizing the presence of linoleic and linolenic fatty acids (18:2 and 18:3). Another way to modify the oil is hydrogenation process. The hydrogenation process is commonly used today because it is easy and cheap; however, it leads to the formation of undesirable nutritionally trans fatty acids [20,21].

Another way to protect the oils and phytosterols during the storage and thermic process may be the use of antioxidants. Antioxidants in oil can occur naturally, or they can be added. One of the most popular antioxidants which occur naturally in oils are tocopherols. The highest levels of tocopherols are observed in crude oil, but the next steps of refining process lead to a reduction of tocopherols content in the oil [22]. Tabee et al. [14] observed the effects of addition of 0.2% α-tocopherol to refined olive oil to stabilization phytosterol during frying. After 12 h of frying, the content of POP was almost 30% lower compared with oil heating without the addition of antioxidants. High protective activities of tocopherols during heating purified triacylglycerols, with the addition of phytosterols, were also observed by other authors [23,24]. Kmiecik at al. [25] investigated the possibility of using natural and synthetic antioxidants (tocopherols and polyphenols) in the stabilization of phytosterols, which were dissolved in triacylglycerols obtained from rapeseed oil, and heated at 180 °C. In all samples with antioxidants, at the end of heating, lower levels of POPs compared to the sample without the addition were observed. However, the final content of POP was dependent on the type of additive. Authors concluded that mixed antioxidants obtained from natural sources (mix of tocopherols and mix of phenolic compounds) have better antioxidants properties because not only a type of antioxidants are important but also the composition and interactions between individual compounds of mixture are crucial.

The aim of the study was to determine how the degree of unsaturation of rapeseed oil and the high temperature of heating process affects the oxidation of naturally occurring phytosterols in rapeseed oil.

## 2. Materials and Methods

### 2.1. Materials

The four oils used in the research were obtained from one production line of hydrogenated rapeseed oil, as the next step of production. The first sample was taken after pressing the seeds (pressed oil), and then refining the oil (refined oil) and during the hydrogenation process (hydrogenated oil). The first sample of hydrogenated oil (iodine value (IV) = 90) was collected during the hydrogenation process (the process was not completed), and the second sample (IV = 79) was a hydrogenated oil collected at the end of the hydrogenation process. All samples were liquid or semi-liquid.

Standards for the identification of phytosterols (β-sitosterol, campesterol, stigmasterol, and brassicasterol) and internal standard (5α-cholestane and 5-cholestane-3β,19-diol) were purchased from Sigma-Aldrich (Sigma-Aldrich, St. Louis, MO, USA) and Steraloids (Steraloids, Newport, RI, USA). Grain fatty acid methyl ester, linoleic acid methyl ester mix, *cis*/*trans,* and linolenic acid methyl ester isomer mix for the identification of fatty acid composition were purchased from Sigma-Aldrich (Sigma-Aldrich, St. Louis, MO, USA). Standards of α-, ß-, γ-, and δ-tocopherols (>95% of purity) were purchased from Merck (Darmstadt, Germany). Sylon BTZ (BSA+TMCS+TMSI, 3:2:3), piridyne, and BSTFA (*N*,*O*-Bis(trimethylsilyl)trifluoroacetamide) with 1% TMCS (trimethylchlorosilane) were purchased from Sigma-Aldrich (Sigma-Aldrich, St. Louis, MO, USA). SEP-PAK amino cartridges were purchased from Waters (Waters, Milford, MA, USA).

### 2.2. Heating Process

Each oil (3.5 L) was heated at 170 ± 5 °C for 48 h (8 h per day, for the following 6 days). The heating process was done in a 3.5 L electric fryer (Stalgast, Poland) and was carried out in triplicate. After every 8 h of heating (1 day), 200 mL of oil was collected, closed under nitrogen, and kept in the freezer at −28 °C, until analyses. Between the next days of heating, the oil was cooled and stored at 5 °C. Before the start of each day of heating, 200 mL of fresh oil was added to the fryer. Phytosterols and oxyphytosterols contents were estimated periodically in no heated oil and after 24 and 48 h of heating.

### 2.3. Phytosterol Content

Phytosterols were analyzed by using the procedure described by Raczyk et al. [26]. Briefly, oil samples (50 mg) with the internal standard (5α-cholestane, 50µL) were saponified with 1 M KOH in methanol, at room temperature, for 18 h. The unsaponifiable fraction was extracted three times with hexane/methyl *tetr* butyl ether (1:1, *v/v*) and after the solvent was evaporated under nitrogen. Sterols were silylated with sylon BTZ and piridyne (Sigma-Aldrich, St. Louis, MO, USA) and analyzed in an Agilent 7820A GC (Agilent Technologies, Wilmington, DE, USA) equipped with a DB-35MS (Agilent Technologies) capillary column (30 m, 0.20 mm, and 0.33 μm). Analysis parameters were as follows: the oven temperature was initially 100 °C, held 5 min, and increased to 250 °C at 25 °C/min., and to 290 °C at 3 °C/min; injector and flame ionization detector (FID) temperature was 250 °C; carrier gas, helium at 1 mL/min. Phytosterols were identified by comparison of their retention times (relative to 5a-cholestane) with commercially available standards.

### 2.4. Phytosterol Oxidation Products (POPs) Content

Phytosterol oxidation products (POPs) were analyzed by using the procedure described by Raczyk et al. [26]. Briefly, oil samples (200 µL) with an internal standard (5-cholestane-3β,19-diol, 500 mg) were transesterificated with a mixture of sodium methylate and methyl *tert*butyl ether (MTBE) (4:6 *v/v*). The oxyphytosterol fraction was extracted with chloroform and dried under nitrogen. After drying, the residue was transferred by chloroform to a SEP-PAK NH2 cartridge (Waters, Milford, Ma, USA) conditioned with 2.5 mL hexane. The column was washed with 2.5 mL hexane, 5 mL hexane/MTBE (5:1 *v/v*), and 5 mL hexane/MTBE (3:1 *v/v*). Then, the fraction of oxyphytosterols was eluded with 7 mL acetone and dried under nitrogen. After evaporation, the samples were silylated with BSTFA with 1% TCMS (Sigma-Aldrich) and analyzed in an Agilent 7820A GC (Agilent Technologies Wilmington, DE, USA) equipped with a DB-35MS (Agilent Technologies) capillary column (30 m, 0.20 mm, and 0.33 μm) and an FID (flame ionization detector). The initial oven temperature was 260 °C for 20 min; then it increased at 0.5 °C/min to 275 °C, followed by 3 °C/min to 290 °C. The injector and detector temperatures were 300 and 310 °C. The samples were injected in splitless mode. The carrier gas was helium, with a constant flow of 1 mL/min. POPs were identified on a 7890A GC system (Agilent Technologies) coupled to a 5975C VL Triple-Axis mass detector (Agilent Technologies), using the column described above. To identify the compounds, a combination of NIST Mass Spectra Library, our laboratory library of collected oxyphytosterol data, and the retention data of the standards was utilized.

### 2.5. Peroxide Value

The peroxide value was determined according to International standard method PN-EN ISO 3960:2017-03 [27].

### 2.6. Anisidine Value

The anisidine value was determined according to International standard method PN-EN ISO 6885:2016-04 [28].

### 2.7. Acid Value

The acid value was determined according to International standard method PN-EN ISO 660:2010 [29].

### 2.8. Total Polar Compounds

Total polar compounds (TPCs) were analyzed according to the American Oil Chemists’ Society (AOCS) Official Method 982.27 [30]. Briefly, analysis was performed in a glass column packed with silica gel (Sigma- Aldrich, silica gel 60, 63–200 µm) conditioned with a mixture of petroleum ether and diethyl ether (87:13 *v/v*). Oil sample was dissolved in eluent solvent and separated in a silica column. Non-polar fractions were eluted by the mobile phase, while polar substances were absorbed on the silica gel. The amounts of polar and non-polar fractions were determined by the weight after evaporation of the solvents. The results were expressed as % of the total content of the oil sample.

### 2.9. Iodine Value

The determination of the iodine value (IV) was conducted according to the AOCS Official Method Cd 1c-85 [31] and calculated (CIV) from fatty acid composition.

### 2.10. Tocopherol Content

The tocopherols contents were determined according to Siger et al. [32]. Briefly, oil was dissolved in hexane and transferred to vials for analyses. The tocopherols contents were analyzed by using Waters HPLC system (Waters, Milford, MA) equipped with a LiChrosorb Si 60 column (Merck, Darmstadt, Germany) (250 mm, 4.6 mm, and 5 µm) and a fluorimetric detector (Waters 474) and a photodiode array detector (Waters 2998 PDA). The mobile phase was a mixture of hexane with 1,4-dioxane (96:4 *v/v*). The flow rate was 1.0 mL/min (for tocopherols and PC-8). To detect the fluorescence of tocopherols, the excitation wavelength was set at ʎ = 295 nm, and the emission wavelength was set at ʎ = 330 nm.

### 2.11. Fatty Acid Composition

The fatty acid composition was determined according to AOCS Official method Ce 1h-05 [33]. Briefly, oil samples (10 mg) were dissolved in hexane and transesterified with sodium methylate (0.1 M). Fatty acid methyl esters (FAMEs) were analyzed by using an Agilent 7820A GC (Agilent Technologies, Santa Clara, CA) equipped with SLB-IL111 capillary columns (Supelco, Bellefonte, PA, USA) (100 m, 0.25 mm, and 0.20 μm) and an FID (flame ionization detector). The oven temperature was initially 150 °C and increased to 200 °C at 1.5 °C/min. The injector and detector temperature was 250 °C and split as 1:10. The carrier gas was helium, at 1 mL/min. The fatty acids (FAs) were identified by comparison of their retention times with commercially available standards.

### 2.12. Statistical Analysis

All assays were performed in three replications. Values of means and standard deviations were calculated with the use of Microsoft Office Excel 2019 (Microsoft, Redmond, WA). STATISTICA PL 13.0 (StatSoft, Inc., Poland) was the software used for principal component analysis (PCA), and to calculate significant differences between means (*p* < 0.05, analysis of variance ANOVA), we used Tukey’s multiple range test.

## 3. Results and Discussion

### 3.1. Chemical Composition of Not Heated Rapeseed Oil

The composition of unheated oils which were used for heating was typical for the various types of rapeseed oil, and it is presented in Table 1. The pressed oil was characterized by an elevated level of peroxide value (1.00 mEq O_2_/kg) and acid value (2.31 mg KOH/g). The refining and hydrogenation process led to a reduction of these indexes. The peroxide value was 0.19, 0.20, and 0.11 mEq O_2_/kg in refined and the first (IV = 90) and the second (IV = 79) partially hydrogenated oil, respectively. The acid value after refining and hydrogenation decreased to 0.15, 0.28, and 0.18 mg KOH/g of oil in oil from the next step of process. The iodine value for pressed and refined oil was at the same high level, 118 g I_2_/100 g of oil. For hydrogenated oils, the iodine value was lower and amounted to 90 and 79 for first and second steps of the hydrogenation process. The lowest level of total polar components was found in refined oil (1.5%). In other oils, TPC was above 6%. The composition of fatty acid in pressed and refined oil was almost the same. Both oils were characterized by a high content of oleic acid (60.28%), linoleic acid (19.14%), and linolenic acid (12.15%). The hydrogenation process of the oil led to the modification of fatty acid composition in the selected oils. In both partially hydrogenated oils, we observed an increase in the content of saturated fatty acids, particularly stearic acid and monounsaturated fatty acid (oleic acid). In the second partially hydrogenated oil (IV = 79), the level of stearic acid was almost 10 times higher, compared to pressed or refined oil and amounted to 11.36%. In these oils, also the high decrease in polyunsaturated fatty acids (PUFA) was observed. In the first partially hydrogenated oil (IV = 90), the level of PUFA was 8.51% and 2.7% for linoleic and linolenic acid, respectively. Further hydrogenation process reduced levels of these acids to 5.78% and 1.89%. In addition to an increase in the content of saturated fatty acids, an increase in the content of trans-fatty acids (TFA) was also observed. In both hydrogenated oil samples, trans-fatty acids constitute from 33.81% (IV = 90) to 39.45% (IV = 79) of the total content of FA. The increase of TFA is typical during partially hydrogenation of refined oil, but level of trans fatty acids in hydrogenation oils depends on various factors, such as temperature and time of hydrogenation, hydrogen pressure, and catalyst type. The high level of trans fatty acid also was observed during the hydrogenation of soybean oil. Prolongation of the time of hydrogenation process led to a rapid increase of the level of trans fatty acid, especially in the second half of the process (from 50 to 100 min), with a relatively lower increase of saturated fatty acids [34]. The total content of tocopherols ranged from 75.19 to 34.49 mg/100 g of oil. The highest level of tocopherols was characteristic for pressed oil. The tocopherol content was systematically decreased in the next stages of oil production. In refined and partially hardened oil (IV = 90 and IV = 79), tocopherols were lower by 13.66, 21.10, and 54.12%, respectively. In all oil samples, the main tocopherols were α- and γ-tocopherol, which together accounted for over 98% of all tocopherols.

### 3.2. Phytosterols Contents in Rapeseed Oil

The content of phytosterols was varied and depends on the type of used oil (Table 2). The highest level of phytosterols was observed in unheated pressed rapeseed oil (9.09 mg/g of oil). The process of refining and hydrogenation led to a reduction in the content of sterols in the oil. The total amount of phytosterols in refined and partially hydrogenated oil (IV = 90 and IV-79) was 6.24, 5.11, and 4.14 mg/g, respectively. In all oil samples, the main sterols were β-sitosterol and campesterol, which together constitute from 70% to 85% of the total sterols in oil. During the heating process, there was a steady decrease in the content of individual and total content of sterols in each of the used oils. The lowest decrease of content of phytosterols was characteristic for the first partially hydrogenated oil (IV = 90), and the pressed oil and amounted to 21.16% and 24.53% respectively. Higher decreases were observed for refined oil (32.37%) and the second hydrogenated oils (IV = 79, 46.14%).

The decrease of phytosterols content in the oil and products enriched with phytosterols is typical during storage and use of fats in thermal processes. This phenomenon is dependent on the process’s temperature, time, composition of fatty acids, and type of phytosterols present in the food (free, esterified) [35,36]. Oehrl et al. [35], during 20 h of heating oil with different fatty acid compositions, at temperatures of 150 and 180 °C, observed very drastic losses of β-sitosterol and campesterol. For refined regular rapeseed oil, the losses were more than 90% of the original content.

A decrease of the process temperature to 100 °C limits the loss of both compounds to 33%. Large differences between the cited work and the results presented in this study may result from conditions of the heating process and especially the ratio between the surface and volume of heated oil. The authors of Reference [35] used for heating a metal pan, and they probably heated a small volume of oil with a relatively large surface area. Deep-frying, as opposed to pan-frying, is a process where a large volume of oil with a relatively small surface contact of oil with the air is used. The obtained data show that the size of phytosterols loss during thermal processes may also depend on the type of process (pan- or deep-frying).

### 3.3. Phytosterols Oxidation Products (POP) Contents in Rapeseed Oil

The high temperature and long heating time lead to the oxidation of phytosterols and the formation of phytosterols oxidation products in each of the used oils. The level of POPs was varied and depends on the type of oil used.

Our analysis of the oxidized derivatives of β-sitosterol showed that the lowest level of these compounds in unheated second partially hydrogenated oil (IV = 79) was observed (Table 3). The content of β-sitosterol oxidized products was 95.71 µg/g of oil. For the other unheated oils, the content of oxidized derivatives of β-sitosterol was higher and amounted to 112.33, 128.18, and 166.60 µg/g of oil for first partially hydrogenated oil (IV = 90), pressed oil, and refined oil, respectively. During 48 h of heating, the content of β-sitosterol oxidation products increased and ranged from 3.12 to 4.7 times. The lowest increase of those compounds was observed in refined (3.12 times) and the first partially hydrogenated oil (IV = 90; 3.8 times). In pressed and the second partially hydrogenated oil (IV = 79), the increase was 4.7 times. In heated pressed oil, the highest increase of β-sitosterol oxidation products was observed in the second part of the heating process. It was almost 4 times higher compared to first 24 h of heating. In other oils, the increase of those compounds was almost the same or only a bit higher in one of the parts of process. Slow increase of β-sitosterol oxidation product in the pressed oil during the first 24 h of heating probably can be explained by the presence of tocopherols. Tocopherols are present at high levels in pressed oils and are reduced at the next steps of the refining process. Tocopherols also undergo rapid degradation under high temperatures, losing its protective function [37]. Pressed oil was also characterized by the highest level of avenasterol and β-sitosterol, which, in some studies, were indicated as substances with a protective effect in heated oil [38,39].

In unheated oils, the main group of β-sitosterol oxidation products were 7α- and 7β-hydroxy sterol, which together accounted for 50% or more of the total content of β-sitosterol derivatives. In pressed oil and the second partially hydrogenated oil (IV = 79), 7α- and 7β-derivatives were at similar level and were 36.7, 34.15, 29.42, and 23.94 µg/g of oil, respectively. In refined oil and the first partially hydrogenated oil (IV = 90), 7β-hydroxy derivatives were at a higher level, compared to 7α-hydroxy, and were 64.8% and 73.6% of these compounds, respectively. At a high level, the presence of 7-keto (39.29 µg/g) in refined oil and derivatives 5β,6β-epoxy β-sitosterol (33.41 µg/g) in the second of hydrogenated oils (IV = 79) was also observed. The heating process leads most often to changes of the profile of the oxidation products of β-sitosterol in analyzed samples. During the heating of pressed oil, a sharp increase of 5α,6α-epoxy and 5β,6β-epoxy derivatives of β-sitosterol was observed. After 48 h of heating, the contents of those compounds amounted to 167.83 and 193.81 µg/g of oil. A very high increase of 5β,6β-epoxy β-sitosterol was also observed in the refined and the first of the partially hydrogenated oil (IV = 90). The content of 5β,6β-epoxy at the end of the heating process was 113.69 and 106.14 µg/g of oil, respectively. After heating in refined oil, the high content of 7β-hydroxy (130.95 µg/g) and 7-keto derivatives (11.39 µg/g) was also observed. In both partially hydrogenated oils, heating also led to an increase in the content of 7-hydroxy derivatives of β-sitosterol, which ultimately accounted for about 50% of the total oxidized derivatives.

The total content of the campesterol oxidation products was lower in all used oils compared to the content β-sitosterol oxidation products, and in unheated oils, it ranged from 42.71 to 76.77 µg/g of oil (Table 4). The lowest content of these compounds was observed in the first hydrogenated oil (IV = 90) and pressed oil. In the refined and the second hydrogenated oil (IV = 79), the content of campesterol oxidation products was almost two times higher, compared to previous oil. During the 48 h of heating, a steady increase in the content of these compounds in the analyzed samples was observed. The lowest increase was characteristic for refined oil, and it was 3.5 times. In other cases, the increase was 5.5, 6, and 6.6 times for the second (IV = 79) and the first (IV = 90) partially hydrogenated oil, and pressed oil, respectively. The final content of campesterol oxidation products ranged from 258.84 to 422.50 µg/g of oil. Compared to the results presented above for β-sitosterol, the increase of campesterol oxidation product was steady in both analyzed parts of the heating process (pressed and second partially hydrogenated oil (IV = 79)) or much higher in the second half of the process (refined and first partially hydrogenated oil (IV = 90)). Our analysis of the composition of campesterol oxidation products in unheated oil showed that the most frequently dominated components were 7α-hydroxy and 7β-hydroxy products in pressed oil; 5β,6β-epoxy, 7α-hydroxy, and 7-ketosterols in refined oil; and 7β-hydroxy in first (IV = 90) and second (IV = 79) partially hydrogenated oil. During the heating, the increase in the contents of each oxidized derivatives of campesterol and changes in the composition of total content were observed. After heating in pressed oil, the dominant groups of oxidation products were 5β,6β-epoxy and 7-keto campesterol, and their content amounted to 76.72 and 68.23 µg/g of oil, respectively. Moreover, 5β,6β-epoxy campesterol was also the dominant group in the refined oil (69.51 µg/g of oil).

In heated partially hydrogenated oils, as well as in unheated oil, the dominant derivatives were 7α- and 7β-hydroxy campesterol. In the first of the hydrogenated oils (IV = 90), the content of these compounds amounted to 40.31 and 77.58 µg/g. In the second hydrogenated oil (IV = 79), the content of these compounds was almost twice as high and amounted to 94.26 and 137.45 µg/g of oil.

The content of stigmasterol oxidation products in unheated oils ranged from 0 to 17.15 µg/g of oil (Table 5). The total absence or very small amount of those components (1.98 µg/g) was observed in pressed oil and first hydrogenated oils (IV = 90), respectively. In the second hydrogenated oil (IV = 79), the amount of oxidized derivatives of stigmasterol was 10.62 µg/g, and for refined oil, it was 17.15 µg/g of oil. During the heating, the content of stigmasterol oxidation products increased from a few to several times, depending on the used oil. After 48 h of the heating process, the content of those components ranged from 41.81 to 128.76 µg/g of oil. During the heating of pressed oil, the increase only of oxidized derivatives of 7α- and 7β-hydroxy stigmasterol was observed. The final levels of those components were 25.76 and 16.13 µg/g of oil, respectively. In other oils, we observed an increase in all of the analyzed oxidation products. For the first hydrogenated oils (IV = 90), a three-times-higher increase of the compounds was observed in the first half of the process, compared to the second part. In other samples, the highest increase was characteristic of the second half of the heating process. Just as previously observed in unheated oils, the main groups of stigmasterol oxidation products were 7α- and 7β-hydroxy stigmasterol. In the heated oils, derivatives of 7α- and 7β-hydroxy (pressed and refined oil) or derivatives of 5α,6α- and 5β,6β-epoxy stigmasterol (the first hydrogenated oil IV = 90) mostly dominated. In the second hydrogenated oil (IV = 79), after the heating process, hydroxy and epoxy derivatives were at a similar level, which ranged from 10.24 to 11.19 µg/g of oil.

The total content of POPs in all oils after 48 h of the heating process was at a similar level. The lowest POPs content was observed in the refined oil and the first of hydrogenated oils (IV = 90), which amounted to 853.07 and 859.29 µg/g of oil, respectively. In pressed oil and the second hydrogenated oil (IV = 79), the level of POPs was slightly higher and amounted to 958.34 and 925.9 µg/g of oil, respectively. The presence of phytosterols oxidation products and their growth is observed in many foods, both stored at refrigerated conditions and subjected to high temperatures. However, one of the main factors speeding phytosterols oxidation in food is the temperature and time of heating operation. Soupas et al. [36], heating rapeseed oil enriched with phytosterols in the pan, observed the rapid increase of oxidized derivatives of β-sitosterol. Heating rapeseed oil at 160 °C for 10 min leads to a 10 times increase in the content of these compounds. Increasing the temperature to 180 °C in the same time leads to 50 times increase in β-sitosterol oxidation products. The authors also note that the form of phytosterols occurring in used oil (free or esterified) is very important. The addition of phytosterol esters to the oil compared to addition of free phytosterols leads to about 40–50% lower level of oxygenated derivatives of β-sitosterol, at the same time and temperature of heating process. This is important due to the fact that the phytosterols present in the oils are in esterified form up to 60% of the total of their contents [40]. This phenomenon can be explained by the structure of phytosterols ester molecules, which somehow must create a physical or steric barrier for oxidation, where oxygen attacks the fatty acid first, slowing the attack on the sterol. When the fatty acids are saturated, the oxygen attacks directly the sterol [41]. According to this theory, the content of POPs in hydrogenated oil (more saturated fat) can be higher compared to pressed and refined oil. However, a detailed analysis of the fatty acid composition of tested oils showed that, despite different iodine values of oils, total content of unsaturated fatty acid was very similar. In pressed, refined, and first hydrogenated oil (IV = 90), the level of unsaturated fatty oil ranged between 90.5% and 93%. In second hydrogenated oil (IV = 79), the unsaturated fatty acid content was 81.5%. However, in hydrogenated oils, the changes in the ratio between oleic, linoleic, and linolenic acid were observed. In these oils, the increase of oleic acid content and decrease of the level of polyunsaturated fatty acids were observed. This may explain the lower content of POPs in hydrogenated oils compared to refined oil. Oehrl et al. [35] noted that a higher PUFA content in oil can accelerate the oxidation of fatty acids; moreover, the accumulation of oxidation products and other soluble substances in the oil can also accelerate the oxidation of fatty acids. Moreover, lower content of POP in the more saturated oils may be also a result of rapid degradation created oxysterols as a result of other processes in the oil. Sterols under the influence of high temperature are not only oxidized. During the heating of sterols, fragmented phytosterols, volatile compounds, and oligomers can be also observed [42].

### 3.4. Principal Component Analysis (PCA)

**Figure 2:**
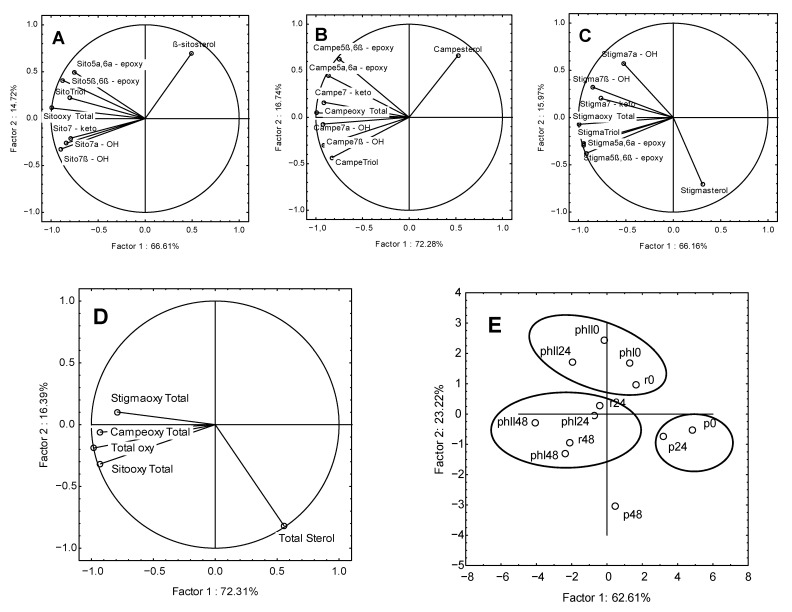
Principal component analysis (PCA) of the loading plot and the score plot of data from ß-sitosterol (**A**), campesterol (**B**), stigmasterol (**C**), and total sum (**D**,**E**) of oxidation products created during heating rapeseed oil at 170 °C. Names of oil sampes: p—pressed, r—refined, phI—partially hydrogenated IV = 90, phII—Partially hydrogenated IV = 79; 0—unheated oil, 24—oil after 24 h of heating, 48—oil after 48 h of heating.

Principal component analysis (PCA) was applied to observe the possible clusters in samples of heated rapeseed oil received from different steps of production. In all loading plots of PCA (Figure 2), the first two principal factors are between 81.33% and 88.7% of the total variation. The PCA results showed differences between the samples of rapeseed oil with different degrees of unsaturation heated at 170 °C. In Figure 2A–C, a strong negative correlation between the first factor and all oxidation products of the individual phytosterols was observed. In Figure 2A, where the oxidized derivatives of ß-sitosterol are presented, the highest negative correlation was characteristic for the sum of oxidized derivatives (−0.990), and then for 7ß-OH (−0.893) and 5ß,6ß-epoxy (−0.876). For oxidized derivatives of campesterol (Figure 2B), the highest negative correlation was observed for the sum of oxidized derivatives (−0.997), and then for 7α-OH (−0.922), 7ß-OH (−0.917), and 7-keto (−0.912). For oxidized derivatives of stigmasterol (Figure 2C), the highest negative correlation was also observed for the sum of oxidized derivatives (−0.993), and then for triols (−0.950), 5α,6α-epoxy (−0.945), and 5ß, 6ß-epoxy (−0.914). In Figure 2D, a correlation for sum of sterols and sum of POPs in the tested samples was observed. Factor 1 was mainly negatively correlated with the content of total POPs (−0.979), total content of oxidized derivatives of ß-stigmasterol (−0.928), and the total content of oxidized derivatives of campesterol (−0.928). Factor 2 was mainly negatively correlated with the content of total sterols (−0.818). The data shown in the score plot (Figure 2E) divided all samples into three main groups. In the first group, located to the right of the *y*-axis, there are two pressed oil samples (unheated oil and heated for 24 h). These samples were characterized by a high content of sterols, 9.09 and 8.09 mg/g of oil, respectively, and a low content of POPs (174.84 and 358.04 µg/g). The second group contains four oil samples (unheated refined oil, both unheated hydrogenated oils and the second partially hydrogenated oil—IV = 79 after 24 h of heating), which were characterized by low or medium POPs content (157.02–447.05 µg/g of oil). The last group contains five samples with moderate or low levels of sterols and high levels of POP, ranging from 534.55 to 925.90 µg/g of oil. A sample of pressed oil heated for 48 h was located outside the presented groups, with a long distance from them. This sample is located at the bottom of the score plot and has the highest POPs content of all analyzed samples (958.34 µg/g of oil) and a high sterol content (6.68 mg/g of oil).

Additionally, the *y*-axis divided the samples into two groups. On the right side of the axis, there were samples with the content of sterols from 5.11 to 9.09 mg/g of oil. On the left side of the *y*-axis, the content of sterols ranged from 2.23 to 5.01 mg/g of oil. As the distance from the axis increased, the content of sterols increased (on the right side) or decreased (on the left side).

## 4. Conclusions

Heating oils of varying degrees of unsaturation led to a rapid increase in the level of phytosterols’ oxidation products in all analyzed samples. Despite differences in content and composition of single phytosterols’ oxidized derivatives, the total content of POP after 48 h of heating was on a similar level for each of the tested oils. This showed that the fatty acid composition of oil is not the only factor that affects the oxidation of phytosterols in foods during heating. Other important factors may also be the presence of other native substances present in the oils as antioxidants or forms of occurrence phytosterols. The hydrogenation process which is used to improve the oxidative stability of the oils in industry did not contribute substantially to reduce the amount of POP generated during heating. However, during the next steps of the refining and hydrogenation process, the content of sterols found in the oil was considerably reduced.

## Figures and Tables

**Table 1:** Chemical composition of rapeseed oil.

	Pressed Oil	Refined Oil	Partially Hydrogenated Oil (IV = 90)	Partially Hydrogenated Oil (IV = 79)
Peroxide value (mEq O_2_/kg)	1.00 ± 0.15 ^a,^*	0.19 ± 0.04 ^b^	0.20 ± 0.03 ^b^	0.11 ± 0.02 ^c^
Anisidine value	0.08 ± 0.00 ^a^	0.26 ± 0.01 ^b^	5.59 ± 0.22 ^c^	4.00 ± 0.23 ^d^
Acid value (mg KOH/g)	2.31 ± 0.27 ^a^	0.15 ± 0.03 ^b^	0.28 ± 0.03 ^c^	0.18 ± 0.02 ^b^
Total polar components (TPC) (%)	8.5 ± 1.32 ^a^	1.5 ± 0.39 ^b^	7.1 ± 0.62 ^a^	6.4 ± 1.38 ^c^
Iodine value (g I_2_/100 g of oil)	118 ± 2.76 ^a^	118 ± 1.16 ^a^	90 ± 1.15 ^b^	79 ± 1.46 ^c^
Tocopherols (mg/100 g)				
α-T	34.44 ± 0.17 ^a^	30.54 ± 0.46 ^b^	27.10 ± 0.41 ^c^	15.21 ± 0.36 ^d^
ᵝ-T	0.30 ± 0.03 ^a^	0.14 ± 0.01 ^b^	0.13 ± 0.02 ^b^	0.05 ± 0.02 ^c^
ᵧ-T	39.53 ± 0.35 ^a^	33.57 ± 0.47 ^b^	31.20 ± 0.65 ^b^	18.71 ± 0.37 ^c^
δ-T	0.91 ± 0.04 ^a^	0.67 ± 0.05 ^a^	0.88 ± 0.17 ^a^	0.52 ± 0.03 ^b^
Total	75.19 ± 0.31 ^a^	64.93 ± 0.99 ^b^	59.32 ± 0.96 ^c^	34.49 ± 0.38 ^d^
Fatty acid composition (%)				
16:0	4.39 ± 0.04 ^a^	4.45 ± 0.02 ^a^	4.77 ± 0.29 ^a^	5.66 ± 0.69 ^b^
16:1	0.21 ± 0.00 ^a^	0.22 ± 0.01 ^a^	0.08 ± 0.03 ^b^	0.08 ± 0.11 ^b^
18:0	1.69 ± 0.03 ^a^	1.70 ± 0.03 ^a^	4.25 ± 0.24 ^b^	11.36 ± 0.85 ^c^
18:1 *cis*	60.43 ± 0.11 ^a^	60.13 ± 0.32 ^a^	53.93 ± 2.47 ^b^	39.93 ± 1.18 ^c^
18:1 *trans*	nd **	nd	24.63 ± 2.63 ^a^	33.15 ± 1.16 ^b^
18:2 *cis*	18.93 ± 0.10 ^a^	19.32 ± 0.39 ^a^	1.00 ± 0.32 ^b^	0.77 ± 0.06 ^b^
18:2 *trans*	nd	0.03 ± 0.00 ^a^	7.51 ± 1.80 ^b^	5.01 ± 0.54 ^c^
18:3 *cis*	12.24 ± 0.12 ^a^	11.51 ± 0.07 ^a^	1.03 ± 0.32 ^b^	0.60 ± 0.05 ^b^
18:3 *trans*	0.02 ± 0.00 ^a^	0.53 ± 0.08 ^b^	1.67 ± 0.13 ^c^	1.29 ± 0.12 ^d^
20:0	0.58 ± 0.03 ^a^	0.58 ± 0.02 ^a^	0.09 ± 0.00 ^b^	0.86 ± 0.04 ^c^
22:0	0.31 ± 0.02 ^a^	0.33 ± 0.01 ^a^	0.36 ± 0.01 ^a^	0.61 ± 0.04 ^b^
22:1	1.20 ± 0.20 ^a^	1.20 ± 0.14 ^a^	0.69 ± 0.18 ^b^	0.67 ± 0.08 ^b^

Values are means of three determinations ± SD. * Means in the same row followed by different letters indicate significant differences (*p* < 0.05) between samples of each type of oil. ** nd—not detected. IV—iodine value.

**Table 2:** Influence of heating time on the phytosterol contents in rapeseed oil heated at 170 ± 5 °C.

Type of Oil	Heating Time	Brassicasterol	Campesterol	Stigmasterol	β-Sitosterol	Avenasterol	Total
	(h)	(mg/g)					
Pressed	Unheated	0.84 ± 0.07 ^a,^*	2.69 ± 0.19 ^a^	0.36 ± 0.00 ^a^	3.62 ± 0.43 ^a^	1.57 ± 0.02 ^a^	9.09 ± 0.35 ^a^
	24	0.83 ± 0.06 ^a^	2.49 ± 0.26 ^ab^	0.29 ± 0.02 ^ab^	3.36 ± 0.60 ^a^	1.11 ± 0.01 ^a^	8.09 ± 0.64 ^b^
	48	0.78 ± 0.08 ^a^	2.32 ± 0.27 ^b^	0.21 ± 0.01 ^b^	2.52 ± 0.76 ^b^	1.03 ± 0.04 ^a^	6.86 ± 0.75 ^c^
Refined	Unheated	0.70 ± 0.07 ^a^	2.25 ± 0.15 ^a^	0.03 ± 0.01 ^a^	2.98 ± 0.22 ^a^	0.29 ± 0.03 ^a^	6.24 ± 0.43 ^a^
	24	0.55 ± 0.06 ^b^	1.82 ± 0.25 ^b^	0.02 ± 0.00 ^ab^	2.39 ± 0.26 ^b^	0.22 ± 0.03 ^b^	5.01 ± 0.59 ^b^
	48	0.46 ± 0.07 ^c^	1.50 ± 0.22 ^c^	0.02 ± 0.00 ^b^	2.05 ± 0.27 ^c^	0.18 ± 0.03 ^b^	4.22 ± 0.57 ^c^
Partially hydrogenated IV = 90	Unheated	0.48 ± 0.10 ^a^	1.38 ± 0.27 ^a^	0.32 ± 0.01 ^a^	2.31 ± 0.76 ^a^	0.69 ± 0.06 ^a^	5.11 ± 0.49 ^a^
	24	0.47 ± 0.11 ^a^	1.32 ± 0.15 ^a^	0.22 ± 0.00 ^a^	2.24 ± 0.17 ^a^	0.40 ± 0.06 ^b^	4.72 ± 0.33 ^a^
	48	0.37 ± 0.10 ^a^	1.17 ± 0.27 ^a^	0.20 ± 0.02 ^a^	1.97 ± 0.28 ^a^	0.32 ± 0.03 ^b^	4.03 ± 0.51 ^a^
Partially hydrogenated IV = 79	Unheated	0.45 ± 0.06 ^a^	1.41 ± 0.18 ^a^	0.04 ± 0.01 ^a^	2.11 ± 0.20 ^a^	0.13 ± 0.08 ^a^	4.14 ± 0.31 ^a^
	24	0.31 ± 0.07 ^b^	1.00 ± 0.24 ^b^	0.03 ± 0.01 ^ab^	1.56 ± 0.27 ^b^	0.05 ± 0.07 ^ab^	2.95 ± 0.43 ^b^
	48	0.25 ± 0.04 ^c^	0.75 ± 0.05 ^c^	0.02 ± 0.02 ^b^	1.19 ± 0.12 ^c^	0.03 ± 0.06 ^b^	2.23 ± 0.14 ^c^

Values are means of three determinations ± SD. * Means in the same column followed by different letters indicate significant differences (*p* < 0.05) between samples of each type of oil.

**Table 3:** Influence of heating time on β-sitosterol oxidation products’ contents in rapeseed oil heated at 170 ± 5 °C.

Type of Oil	Heating Time	7α-OH	7β-OH	5α,6α-Epoxy	5β,6β-Epoxy	Triol	7-keto	Total
	(h)	(μg/g)						
Pressed	Unheated	36.70 ± 1.06 ^a,^*	34.15 ± 0.99 ^a^	3.52 ± 0.10 ^a^	27.69 ± 0.80 ^a^	nd **	26.12 ± 0.75 ^a^	128.18 ± 3.70 ^a^
	24	43.28 ± 4.14 ^a^	66.77 ± 1.26 ^b^	7.63 ± 1.24 ^a^	71.46 ± 5.51 ^b^	4.96 ± 0.17 ^a^	26.42 ± 1.12 ^a^	220.53 ± 17.09 ^b^
	48	61.20 ± 1.91 ^b^	88.54 ± 2.90 ^c^	167.83 ± 5.11 ^b^	193.81 ± 2.06 ^c^	26.29 ± 2.88 ^b^	69.73 ± 1.21 ^b^	607.40 ± 7.85 ^c^
Refined	Unheated	29.45 ± 1.86 ^a^	54.23 ± 5.67 ^a^	14.33 ± 1.31 ^a^	21.40 ± 0.14 ^a^	7.90 ± 0.22 ^a^	39.29 ± 2.54 ^a^	166.60 ± 6.66 ^a^
	24	60.68 ± 1.77 ^b^	95.62 ± 3.35 ^b^	34.26 ± 1.42 ^b^	86.15 ± 0.01 ^b^	11.64 ± 1.02 ^ab^	91.78 ± 2.49 ^b^	380.13 ± 0.20 ^b^
	48	79.68 ± 2.69 ^c^	130.95 ± 1.84 ^c^	69.04 ± 2.33 ^c^	113.69 ± 0.99 ^c^	15.01 ± 1.21 ^b^	111.39 ± 3.77 ^c^	519.77 ± 8.43 ^c^
Partially hydrogenated IV = 90	Unheated	18.38 ± 0.65 ^a^	51.31 ± 0.02 ^a^	13.81 ± 2.05 ^a^	13.13 ± 1.10 ^a^	nd	15.70 ± 1.80 ^a^	112.33 ± 0.22 ^a^
	24	77.43 ± 1.48 ^b^	82.20 ± 1.79 ^b^	19.83 ± 2.07 ^a^	87.25 ± 7.98 ^b^	5.40 ± 0.35 ^a^	26.90 ± 1.76 ^b^	299.01 ± 7.90 ^b^
	48	111.09 ± 3.66 ^c^	118.64 ± 1.03 ^c^	39.15 ± 2.51 ^a^	106.14 ± 3.26 ^c^	40.54 ± 3.26 ^b^	56.13 ± 2.93 ^c^	471.69 ± 7.96 ^c^
Partially hydrogenated IV = 79	Unheated	29.42 ± 1.78 ^a^	23.94 ± 1.53 ^c^	0.90 ± 0.07 ^a^	33.41 ± 2.49 ^a^	nd	8.03 ± 0.60 ^a^	95.71 ± 3.40 ^a^
	24	37.86 ± 3.19 ^b^	59.83 ± 6.72 ^b^	37.18 ± 1.15 ^b^	42.92 ± 0.90 ^b^	3.90 ± 0.48 ^a^	70.20 ± 2.05 ^b^	251.89 ± 10.74 ^b^
	48	91.68 ± 0.89 ^c^	139.34 ± 1.35 ^c^	43.86 ± 1.95 ^c^	84.55 ± 1.07 ^c^	8.45 ± 0.76 ^b^	83.42 ± 0.81 ^c^	451.29 ± 4.67 ^c^

Values are means of three determinations ± SD. * Means in the same column followed by different letters indicate significant differences (*p* < 0.05) between samples of each type of oil. ** nd—not detected.

**Table 4:** Influence of heating time on campesterol oxidation products’ contents in rapeseed oil heated at 170 ± 5 °C.

Type of Oil	Heating Time	7α-OH	7β-OH	5α,6α-Epoxy	5β,6β-Epoxy	Triol	7-Keto	Total
	(h)	(μg/g)						
Pressed	Unheated	22.76 ± 0.66 ^a,^*	11.09 ± 0.32 ^a^	7.89 ± 0.23 ^a^	4.92 ± 0.14 ^a^	nd **	nd	46.66 ± 1.35 ^a^
	24	23.55 ± 4.72 ^a^	26.75 ± 1.05 ^b^	20.39 ± 1.80 ^b^	27.02 ± 1.72 ^b^	7.67 ± 0.12 ^a^	17.66 ± 0.92 ^b^	123.04 ± 2.18 ^b^
	48	50.13 ± 4.99 ^b^	47.57 ± 2.86 ^c^	48.13 ± 4.26 ^c^	76.72 ± 5.64 ^c^	18.35 ± 0.01 ^b^	68.23 ± 3.30 ^b^	309.13 ± 3.80 ^c^
Refined	Unheated	15.08 ± 0.95 ^a^	10.05 ± 0.99 ^a^	10.11 ± 0.41 ^a^	16.90 ± 1.02 ^a^	3.41 ± 0.57 ^a^	15.71 ± 0.55 ^a^	71.26 ± 0.21 ^a^
	24	25.66 ± 0.59 ^b^	34.46 ± 1.72 ^b^	11.91 ± 0.75 ^a^	26.28 ± 0.44 ^b^	2.34 ± 0.34 ^b^	37.00 ± 1.71 ^b^	137.65 ± 2.81 ^b^
	48	37.22 ± 0.20 ^c^	48.10 ± 0.27 ^c^	43.61 ± 2.71 ^b^	69.51 ± 1.79 ^c^	10.73 ± 0.36 ^c^	45.71 ± 3.55 ^c^	254.87 ± 5.30 ^c^
Partially hydrogenated IV = 90	Unheated	9.67 ± 0.82 ^a^	11.71 ± 1.33 ^a^	4.80 ± 0.78 ^a^	5.73 ± 0.52 ^a^	2.90 ± 0.51 ^a^	7.90 ± 0.91 ^a^	42.71 ± 3.31 ^a^
	24	26.93 ± 0.24 ^b^	33.71 ± 1.75 ^b^	12.28 ± 0.72 ^b^	11.31 ± 1.08 ^b^	25.20 ± 3.45 ^b^	30.53 ± 2.80 ^b^	139.96 ± 3.02 ^b^
	48	40.31 ± 0.81 ^c^	77.58 ± 1.80 ^c^	21.87 ± 1.20 ^c^	29.01 ± 1.26 ^c^	41.90 ± 1.60 ^c^	48.17 ± 5.32 ^c^	258.84 ± 5.21 ^c^
Partially hydrogenated IV = 79	Unheated	16.65 ± 1.89 ^a^	24.77 ± 1.84 ^a^	15.62 ± 1.16 ^a^	17.97 ± 1.34 ^a^	1.10 ± 0.08 ^a^	0.67 ± 0.05 ^a^	76.77 ± 2.59 ^a^
	24	24.68 ± 2.81 ^a^	35.12 ± 2.15 ^a^	20.93 ± 2.03 ^a^	27.58 ± 1.17 ^b^	16.10 ± 1.18 ^b^	42.43 ± 2.36 ^b^	166.85 ± 6.50 ^b^
	48	94.26 ± 1.23 ^b^	137.45 ± 3.92 ^b^	41.96 ± 0.41 ^b^	43.89 ± 0.42 ^c^	43.89 ± 0.42 ^c^	61.03 ± 0.31 ^c^	422.50 ± 3.58 ^c^

Values are means of three determinations ± SD. * Means in the same column followed by different letters indicate significant differences (*p* < 0.05) between samples of each type of oil. ** nd—not detected.

**Table 5:** Influence of heating time on stigmasterol oxidation products’ contents in rapeseed oil heated at 170 ± 5 °C.

Type of oil	Heating Time	7α-OH	7β-OH	5α,6α-epoxy	5β,6β-epoxy	Triol	7-keto	Total
	(h)	(μg/g)						
Pressed	Unheated	nd **	nd	nd	nd	nd	nd	nd
	24	11.82 ± 0.55 ^a,^*	2.65 ± 0.04 ^a^	nd	nd	nd	nd	14.47 ± 0.59 ^a^
	48	25.67 ± 0.02 ^b^	16.13 ± 0.20 ^b^	nd	nd	nd	nd	41.81 ± 2.02 ^b^
Refined	Unheated	6.07 ± 0.62 ^a^	nd	1.30 ± 0.42 ^a^	2.69 ± 0.00 ^a^	2.14 ± 0.31 ^a^	4.95 ± 0.51 ^a^	17.15 ± 0.85 ^a^
	24	8.06 ± 0.08 ^b^	8.02 ± 0.57 ^a^	2.60 ± 0.05 ^a^	3.12 ± 0.13 ^a^	2.63 ± 0.18 ^a^	6.59 ± 0.38 ^b^	31.02 ± 0.95 ^b^
	48	17.65 ± 0.45 ^c^	15.04 ± 0.51 ^b^	12.89 ± 0.44 ^b^	15.96 ± 0.54 ^b^	7.83 ± 0.43 ^b^	9.06 ± 0.09 ^c^	78.43 ± 2.45 ^c^
Partially hydrogenated IV = 90	Unheated	1.98 ± 0.04 ^a^	nd	nd	nd	nd	nd	1.98 ± 0.01 ^a^
	24	9.33 ± 1.42 ^b^	12.33 ± 1.33 ^a^	21.43 ± 1.79 ^a^	36.97 ± 2.11 ^a^	11.19 ± 1.72 ^a^	4.32 ± 0.47 ^a^	95.58 ± 5.74 ^b^
	48	15.92 ± 0.56 ^c^	17.87 ± 0.33 ^b^	27.43 ± 0.94 ^b^	41.11 ± 4.50 ^a^	18.52 ± 0.98 ^b^	7.92 ± 0.81 ^b^	128.76 ± 6.20 ^c^
Partially hydrogenated IV = 79	Unheated	4.13 ± 0.31 ^a^	4.20 ± 0.31 ^a^	1.12 ± 0.08 ^a^	1.17 ± 0.09 ^a^	nd	nd	10.62 ± 0.79 ^a^
	24	6.15 ± 0.15 ^b^	7.08 ± 0.76 ^a^	3.93 ± 0.42 ^b^	7.84 ± 0.07 ^b^	2.74 ± 0.40 ^a^	0.57 ± 0.07 ^a^	28.31 ± 1.58 ^b^
	48	10.24 ± 0.71 ^c^	11.19 ± 1.48 ^b^	11.90 ± 0.11 ^c^	10.64 ± 0.10 ^c^	5.52 ± 0.38 ^b^	2.63 ± 0.03 ^b^	52.11 ± 1.57 ^c^

Values are means of three determinations ± SD. * Means in the same column followed by different letters indicate significant differences (*p* < 0.05) between samples of each type of oil. ** nd—not detected.

## Data Availability

Not applicable.
